# 改进的广义回归神经网络用于基于气相色谱法的原油密度定量分析

**DOI:** 10.3724/SP.J.1123.2021.12001

**Published:** 2022-05-08

**Authors:** Haibo LIANG, Shuai DING, Qi WEI, Jialing ZOU

**Affiliations:** 西南石油大学机电工程学院, 四川 成都 610500; School of Mechatronic Engineering, Southwest Petroleum University, Chengdu 610500, China

**Keywords:** 气相色谱, 广义回归神经网络, 麻雀搜索优化算法, 原油密度, gas chromatogram (GC), generalized regression neural network), sparrow search optimization algorithm, crude oil density

## Abstract

在油气勘探开发领域,快速识别储集层原油性质对于工程技术人员有非常重要的指导意义。地球化学录井技术是用于判断储集层原油性质的常规手段,能为储集层综合评价提供专业认识。该文研究了地化录井中的岩石热解分析和气相色谱分析的原理,提出了一种利用色谱谱图对原油密度进行定量分析的新方法,再结合原油性质密度划分标准,可快速判断储集层原油性质。首先使用计算机图像处理软件对色谱图进行标准化和归一化预处理,并分析了岩石热解气相色谱谱图的曲线特征规律,提出了岩石热解气相色谱谱图的特征参数提取方法,将色谱图转换为特征参数矩阵。其次,研究了3种人工智能预测分类模型,为了适合该实验对储集层原油密度的预测,对其做了部分优化改进,利用元启发式优化算法(麻雀搜索优化算法)对广义回归神经网络的超参数进行了优化,提高了模型的精度和收敛速度。最后,通过现场获得的气相色谱图样本对各模型进行训练并验证,综合对比3种模型预测的结果,发现基于麻雀搜索算法优化的广义回归神经网络预测模型效果最佳,模型稳定,预测密度误差小,可泛化能力强。该研究所提出的方法能为储集层的综合评价研究和现场施工提供可靠的数据支撑。

## Abstract (en)

In the field of oil and gas exploration and development, the quick identification of reservoir crude oil properties has a guiding significance for engineers and technicians. Geochemical logging technology is a conventional method to evaluate the properties of crude oil in reservoirs, and it can provide professional knowledge for comprehensive evaluation of reservoirs. In this study, the principles of rock pyrolysis and gas chromatographic analyses in geochemical logging are studied. Moreover, a new method for quantitative analysis of crude oil density by chromatogram is proposed. Combined with the division standard of crude oil property density, the properties of reservoir crude oil can be quickly evaluated. In the experiment, first, the chromatogram was standardized and normalized using computer image processing software. The curve characteristic law of rock pyrolysis gas chromatogram was analyzed, and the corresponding characteristic parameter extraction method was proposed. The chromatogram was converted into a characteristic parameter matrix. Second, three types of artificial intelligence prediction and classification models were studied. The latest meta-heuristic optimization algorithm (sparrow search optimization algorithm) was used to optimize the hyperparameters of the generalized regression neural network, and the accuracy and convergence speed of the model were improved. To study the influence of different positions of rock samples on the experimental results, two groups of samples were utilized: cuttings samples and wall core samples. Based on a comprehensive comparison of the prediction results of the three models, it was found that the generalized regression neural network prediction model optimized by sparrow search algorithm provided the best effect, being a stable model, with small prediction density error, and strong generalization ability. The prediction error coincidence rate (absolute error < 0.02) of this model for cuttings and wall core samples was 95% and 100%, respectively. The root mean square errors were 0.0079 and 0.0069 respectively. The classification accuracy of crude oil properties was 95%. The analysis of the two groups of parallel experimental data indicated that the rock samples from the wall center can more accurately reflect the crude oil properties of the reservoir. Therefore, the method proposed in this study can provide reliable data support for reservoir comprehensive evaluation and on-site construction.

储集层原油性质的快速判断,对录井油气水解释、电缆测压取样、地层测试工具及工艺的选择、成藏模式研究有着重要作用^[1]^。目前地球化学录井技术能够有效定量描述储集层含油气丰度、烃组分分布状态等,了解储集层原油性质的细微变化、原油遭受破坏的程度,发现地下地质现象和规律的特殊性,为储集层的综合评价研究提供可靠性的认识^[2]^。

岩石热解分析和气相色谱分析是地化录井中主要的技术方法^[3]^。岩石热解分析技术是根据有机质热蒸发或热裂解特性,对含有机质的岩样进行程序升温加热,使其中的烃类(油、气)热蒸发成气体,并使高聚合有机质(干酪根、沥青质、胶质)热裂解为挥发性的烃类产物,通过惰性气体携带走热解产物,以氢焰检测器定量检测;样品内的残余有机碳在600 ℃温度下氧化,生成CO_2_和少量CO,由红外或热导检测器检测,经计算得到残余有机碳的含量^[4⇓-6]^。气相色谱分析包括热蒸发气相色谱、轻烃气相色谱等。热蒸发气相色谱技术是一种应用于岩石中烃类组分检测的录井方法,原理是将样品在热解炉中加热到300~350 ℃,使存在于储集岩孔隙或裂缝中的油气组分挥发,用气相色谱分离这些产物,并通过火焰离子化检测器(flame ionization detector, FID)检测,由计算机自动记录各组分的色谱峰及其相对含量^[4,5]^。为了避免储层原油中较重烃类热裂解成轻烃或烯烃,导致分析的烃类组分分布失真,热蒸发烃分析的温度必须控制在350 ℃以下。轻烃气相色谱技术以原油中的轻烃为研究对象。轻烃泛指原油中的汽油馏分,主要以C_1_~C_2_为主,包括烷烃、环烷烃和芳香烃3族烃类,是石油和天然气的重要组成部分,在原油中含量最高,组分最丰富。

根据储集层原油性质密度划分标准^[1,7]^,在温度20 ℃条件下,将原油分为凝析油(密度≤0.830 g/cm^3^)、轻质油(密度在0.830~0.870 g/cm^3^之间)、中质油(密度在0.870~0.920 g/cm^3^之间)、重质油(密度在0.920~1.000 g/cm^3^之间)、超重油(密度≥1.000 g/cm^3^)。通过计算原油密度直接评价原油性质是一个重要方向。利用岩石热解分析的3个主要参数*S*_0_(90 ℃时检测的单位质量岩石中的烃含量(气态烃), mg/g)、*S*_1_(300 ℃时检测的单位质量岩石中的烃含量(液态烃), mg/g)、*S*_2_(300~600 ℃检测的单位质量岩石中的烃含量,mg/g)以及其派生参数PG(含油气总量,mg/g)、TPI(总产率指数)、OPI(油产率指数)、HPI(原油中重质烃类及胶质和沥青质含量)、PS(轻重烃比),可以建立原油密度的定量评价公式^[4]^,该模型能够较准确地预测储集层原油密度,为试油及开发的措施、方案提供一定的数据支撑。但是该模型利用特定区域的数据建模,模型参数固定,因此模型的多区域泛化能力不强,并且需要岩石热解分析的大量原始数据。通过建立地化(岩石热解、热解气相色谱)衍生参数解释评价图版来对储集层油质类型进行判断也是目前常用的方法,利用热解气相色谱和岩石热解分析中主峰碳数、∑nC_21_-/∑nC_22_+(nC表示正构烷烃,下标表示碳数;∑nC_21_-: nC_21_以前组分质量分数总和;∑nC_22_+: nC_21_以后组分质量分数总和)和(*S*_0_+*S*_1_)/*S*_2_这3个主要参数,组合3种不同种类的解释图版用于判断原油性质,该方法应用于中国渤海油田A区域效果良好,能够较为准确地判断出储集层原油性质^[5,7,8]^,与常规气测录井相比提高了判断的准确率。但该图版的建立需要使用大量实际数据作为基础来计算趋势线并划分不同油质区域,效率较低,且该方法建立的解释评价图版只适用于特定区域,更换不同区域的油井后需要重新建立解释评价图版,因此该方法缺少泛化能力。随着近年来大数据与人工智能的快速发展,许多研究人员也将数据挖掘技术应用在录井原油性质判别中,对岩石热解数据和气相色谱数据进行适当数据处理并提取合适的特征参数,再利用决策树即可对原油性质进行分类^[9]^。相比于以上两种常规分析判断方法,应用该技术对原油性质分类的效率有所提高,但是准确率相对较低,且其训练所用数据量比较少,应用范围也相对较小,尚有很大提升空间。除了以上基于岩石热解和气相色谱数据进行的原油性质判断外,还有基于高斯过程回归预测欠饱和油藏密度预测,以及多层前馈反向传播感知器预测欠饱和原油密度这两种方法,但是该方法只适用于欠饱和油藏,且其尚未直接提出对原油性质的预测分类方法^[10,11]^。

为了适应工程录井的智能化发展方向,减少人力成本的同时加快对储集层原油性质的判断,本文提出一种新的储集层原油密度预测模型,该模型优化了地化录井岩石热解气相色谱图特征参数提取方法,优选了8种与密度相关性较大的谱图特征参数,将其与反向传播神经网络、广义回归神经网络以及麻雀搜索优化算法相结合,实现了对目标储集层原油密度的定量分析,模型收敛速度快,准确率高且具有较强的泛化能力,适用于对不同区域不同储集层原油密度的快速预测。

## 1 实验方法

### 1.1 气相色谱条件

RZF-3000型热解组分仪(山东鲁南瑞虹化工仪器有限公司),Petrocol^®^ EX2887毛细管色谱柱(5.0 m×0.53 mm×0.10 μm,美国默克公司),热解炉口温度300 ℃,火焰离子化检测器温度310 ℃;色谱柱初始柱温100 ℃,以10 ℃/min程序升温至300 ℃,恒温25 min,运行结束。氮气作载气,流速为41.5 mL/min;氢气作燃气,流速为40 mL/min;空气作助燃气和动力气,流速为300 mL/min,分流比为1∶60;尾吹用氮气,流速为25 mL/min。

### 1.2 地化谱图特征的提取

储集层原油中的部分烃总是会与岩石中的细菌和氧气发生菌解、氧化作用^[1]^。该作用会形成一定量的未分辨化合物(unresolved complex mixtures, UCMs),色谱柱无法细分,但能测定其总量^[12]^,在色谱图中的表现是形成基线隆起特征。为了表征这一重要特征,需要计算原油的生物降解度(*D*)^[1]^:


(1) $D=\frac{A_2}{A_1}\times 100\%$

其中*A*_1_代表曲线峰顶连线数值积分,*A*_2_代表曲线谷底连线数值积分。*A*_1_与*A*_2_采用顶峰识别算法,提取特征峰特征,忽略不需要识别的小峰,然后对识别到的峰进行数值积分。

根据岩石热解气相色谱的原理可知,主峰出现时间越靠后碳数越大。同时基于原油生物降解原理,主峰碳数越大生物降解程度越严重。因此本实验将色谱图中最高峰的保留时间作为一个预测原油密度、划分原油性质的重要特征参数。

本文所研究的色谱图中正构烷烃主要分布在nC_12_~nC_37_,其中主峰碳C_17_~C_18_为凝析油,C_21_~C_23_为轻质油,C_22_~C_24_为中质油,C_28_为重质油^[1]^。某一碳数烃的出峰时间并不能完全代表原油性质,但将碳数相近的烃类放在一起便可作为一个重要特征。基于此,为了表征某类烃的出峰特征,本文提出色谱峰面积分割法,将整个谱图峰面积按照某类烃的峰面积进行纵向分割。具体来说,每4个碳数划分一个区域,在时间轴上均匀划分出6个区域。各分割区域的面积即可作为气相色谱不同烃类出峰的特征。

大部分气相色谱图样本的基线位置处曲线特征比较复杂,很难确定,需要特殊处理。中值滤波和高斯平滑处理广泛用于图像降噪处理^[13]^,本实验采用该方法去除色谱基线周围的特征峰,准确确定谱图的基线位置。使用MATLAB软件,调用函数medfilt()对图像处理实现中值滤波,调用smoothdata()函数实现高斯平滑处理。

各特征值与原油密度相关系数*r*按照以下公式^[9]^计算:


(2) $r=\frac{\sum(V-\bar{V})(W-\bar{W})}{\sqrt{\sum (V-\bar{V})^{2}(W-\bar{W})^{2}}}$

其中*V*、*W*分别是需要计算相关性系数的两个参数值, $\bar{V}$ 、 $\bar{W}$ 分别为对应参数的平均值。

### 1.3 模型建立

#### 1.3.1 反向传播神经网络预测模型

反向传播神经网络是一种按照误差逆向传播算法训练的多层前馈神经网络^[14]^。该网络通常有一个或多个隐层,本文在该层中均采用sigmoid型传递函数,为了实现对原油密度的预测所以输出层中传递函数采用线性传递函数,这样整个反向传播神经网络便可以输出任意值。根据前文提取的特征参数,每一张色谱图可以提取8个特征值,组成一个8维特征向量作为反向传播神经网络模型的输入。因此这里将反向传播神经网络的输入层定为8维,输出层定为1维。本文采用网络结构增长型方法选取隐层节点个数,即根据经验公式(3)^[14]^,先设置较少的节点数,对网络进行训练,并测试学习误差,然后逐渐增加节点数,直到学习误差不再明显减少为止。


(3) $n_1=\sqrt{n+m}+a$

式中*n*_1_取整数部分作为反向传播网络的隐层节点个数,*n*为输入节点个数,*m*为输出节点个数,*a*为1到10的常数。

综合考虑单个样本的误差与全局误差,采用均方根误差RMSE来衡量整个网络模型的误差:


(4) RMSE=$\frac{1}{mk}\sum_{i=1}^{k}\sum_{j=1}^{m}(\hat{y}_{ij}-y_{ij})^2$

式中*k*为训练样本数目, ${\dot{y}}_{ij}$ 为期望输出值,*y_ij_*为实际输出值。

#### 1.3.2 基于麻雀搜索算法优化广义回归神经网络预测模型

广义回归神经网络建立在非参数核回归的基础上,是基于径向基函数网络的一种改进神经网络模型^[15⇓⇓⇓-19]^。如图1所示为广义回归神经网络模型结构图,输入层的神经元数目等于学习样本中输入向量的维数,模式层神经元数目等于学习样本的数目,输出层神经元数目等于样本输出向量维数。输入层神经元不进行运算,直接将输入变量*X*传递给模式层。模式层又称为隐回归层,各神经元对应不同的样本,神经元*i*的传递函数为^[15]^:


(5) $P_{i}=\exp \left[-\frac{\left(X-X_{i}\right)^{T}\left(X-X_{i}\right)}{2 \sigma^{2}}\right],(i=1,2, \cdots, n)$

**图1:**
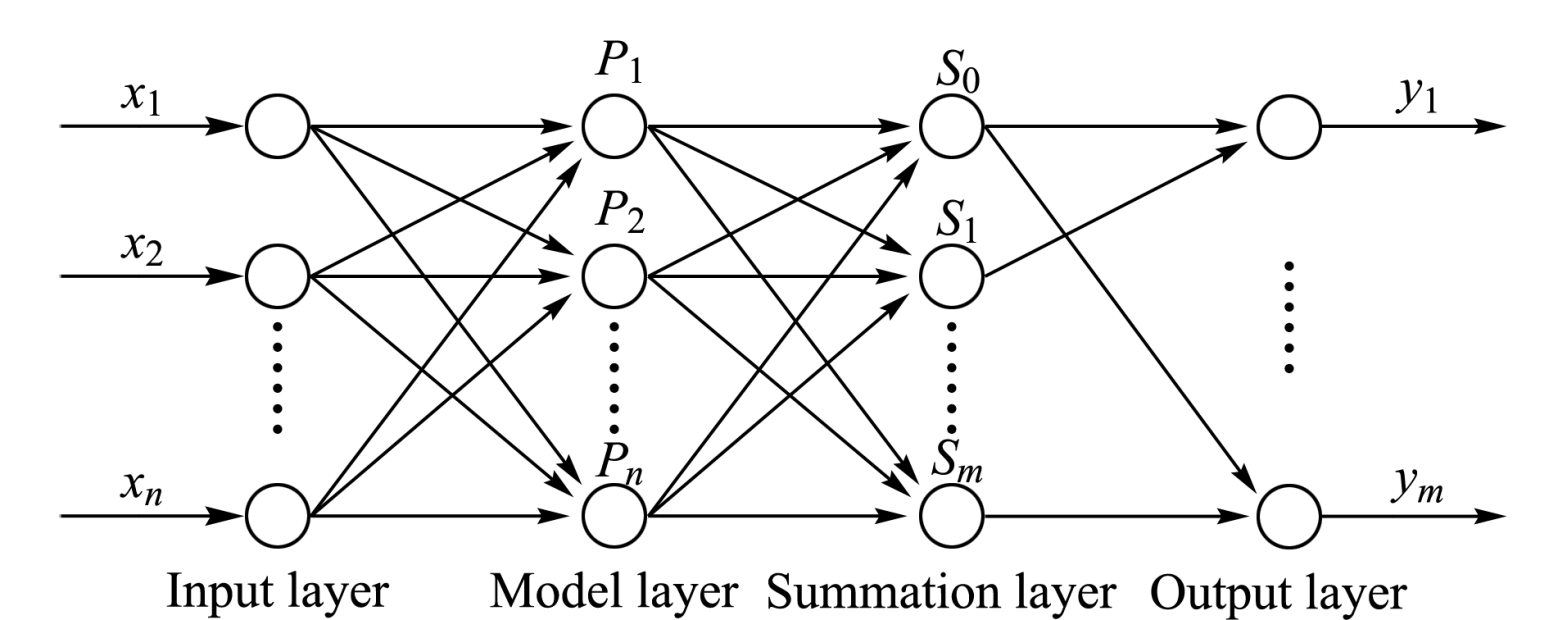
广义回归神经网络结构

其中*σ*为平滑因子。

模式层每个神经单元对应一个训练样本,以高斯核函数为活化核函数。求和层共由两个单元组成,分别求用于输出层计算的分子项和分母项。其中第一个单元与输出节点个数*m*对应,第*j*个节点输出*S_j_*为输出向量*Y*中的第*j*个元素*y_ij_*与模式层中第*i*个神经元输出*P_i_*的加权和,即模式层中第*i*个神经元*P_i_*与求和层中第*j*个神经元间的连接权值为*y_ij_*,其计算公式如下^[16]^:


(6) $\begin{aligned} S_{j}=\sum_{i=1}^{n} y_{i j} p_{i}=& \sum_{i=1}^{n} Y_{i} \exp \left[-\frac{\left(X-X_{i}\right)^{T}\left(X-X_{i}\right)}{2 \sigma^{2}}\right] \\ &(j=1,2, \cdots, m) \end{aligned}$

第2个单元只有一个节点,即模式层各神经元与求和层的连接权值为1,输出*S*_0_为模式层各节点输出*P_i_*之和,其计算公式如下^[17]^:


(7) $S_{0}=\sum_{i=1}^{n} p_{i}=\sum_{i=1}^{n} \exp \left[-\frac{\left(X-X_{i}\right)^{T}\left(X-X_{i}\right)}{2 \sigma^{2}}\right]$

输出层各神经元的输出即估计值为求和层中两个单元的求和结果相除,其计算公式如下^[18]^:


(8) $\hat{Y}_{j}=y_{j}=\frac{S_{j}}{S_{0}},(j=1,2, \cdots, m)$

当训练数据很大时,广义回归神经网络不需要像反向传播神经网络那样进行多次迭代计算,该模型具有更快的学习速度,并能够收敛到最优回归曲面。对于广义回归神经网络算法,当所输入的训练样本集确定时,神经网络训练本质上是确定平滑因子*σ*的过程,网络的预测性能与平滑因子*σ*的取值有很大关联。

受到麻雀的觅食行为和反捕食行为的启发,有研究提出了麻雀搜索算法^[20,21]^,该算法能在限定范围内快速搜索最佳参数,获得最优解。本文使用该算法优化广义回归神经网络的平滑因子*σ*。模型输入特征向量同上文,设定*σ*的搜索空间为(0, 2),以及麻雀搜索算法的适应度函数为广义回归神经网络模型训练均方根误差RMSE:


(9) f(σ)=RMSE_GRNN_

## 2 结果与讨论

### 2.1 实验样本

现场钻井施工得到的240个岩石样本中,有120个样本是岩屑取样,另外120个是壁心取样,经过岩石热解气相色谱分析得到240个谱图样本,其中各类样本所占比重如图2所示。岩屑取样和壁心取样得到的是来自不同位置的岩石样本,因此其经过岩石热解气相色谱分析得到的样本谱图特征有一定差异。在训练模型时,这两种样本应独立分析对比,以验证最佳取样位置,提高数据来源的可靠性。

**图2:**
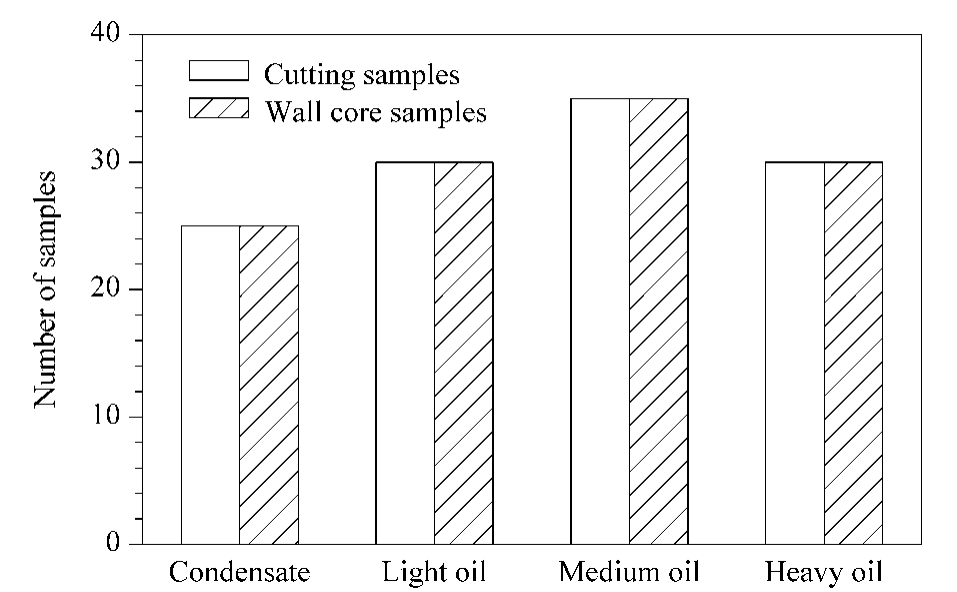
原始谱图样本分类

### 2.2 气相色谱图处理结果

按照1.1节的方法提取色谱图样本的各项特征参数,图3是计算降解度时色谱处理结果图,在MATLAB软件中使用顶峰识别算法获取色谱图的峰顶和谷底并将其连接为封闭图形,图中曲谷底数值积分面积*A*_2_远远小于曲线峰顶数值积分面积*A*_1_,根据公式(1)可知,降解度*D*更接近0,反映出储集层原油生物降解度低。

**图3:**
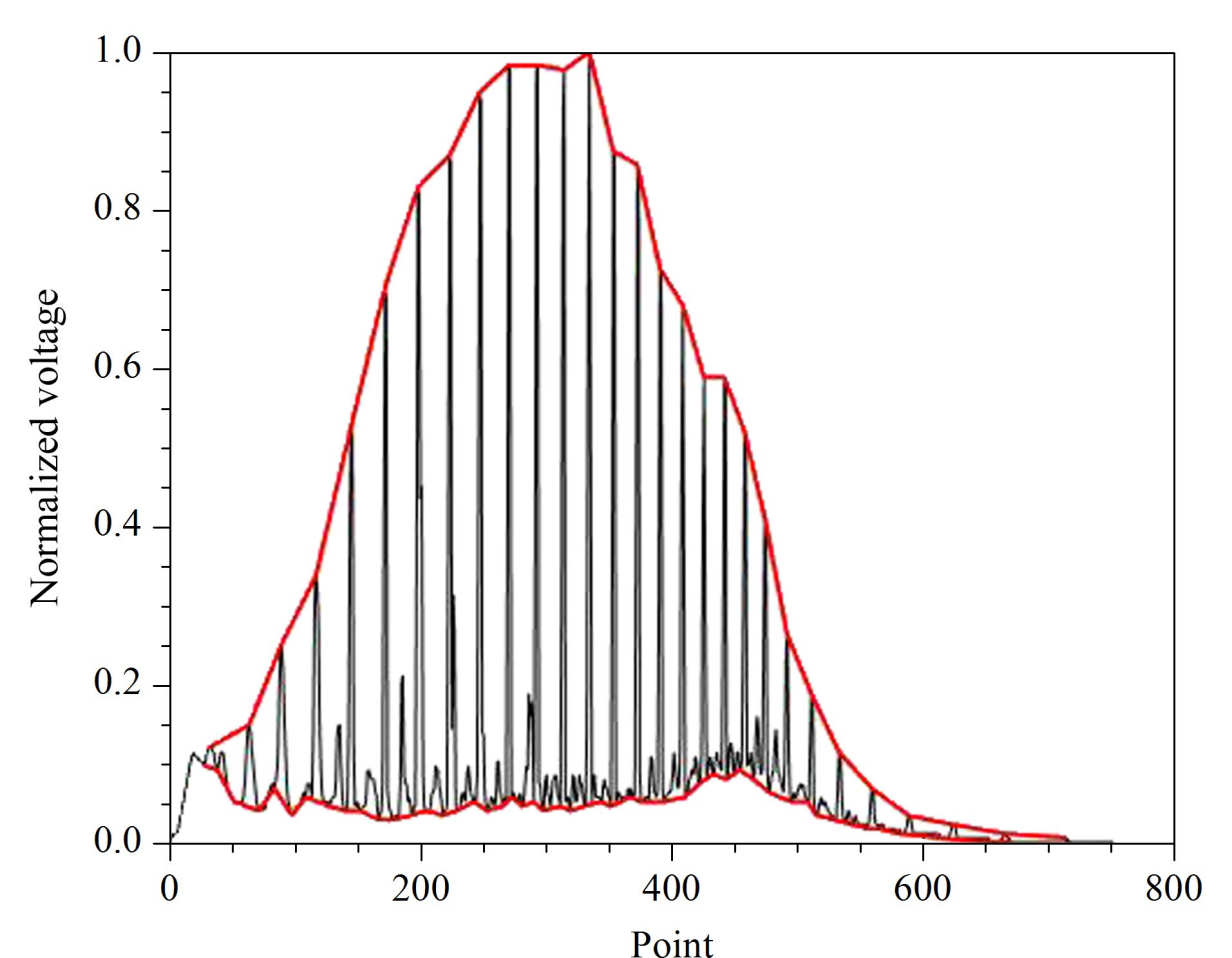
顶峰连线、谷底连线处理图

中值滤波对脉冲噪声有良好的滤除作用,特别是在滤除噪声的同时,能够保护信号的边缘,使之不被模糊,这些优良特性是线性滤波方法所不具有的。高斯滤波是一种线性平滑滤波,适用于消除色谱图中的高斯噪声。图4为滤波后的谱图曲线以及计算的基线位置,与图5对比可以看出,基线位置并不是谷底连线,一般基线位置高于谷底连线。这里确定的基线位置可以用于后续计算基线分区面积。

**图4:**
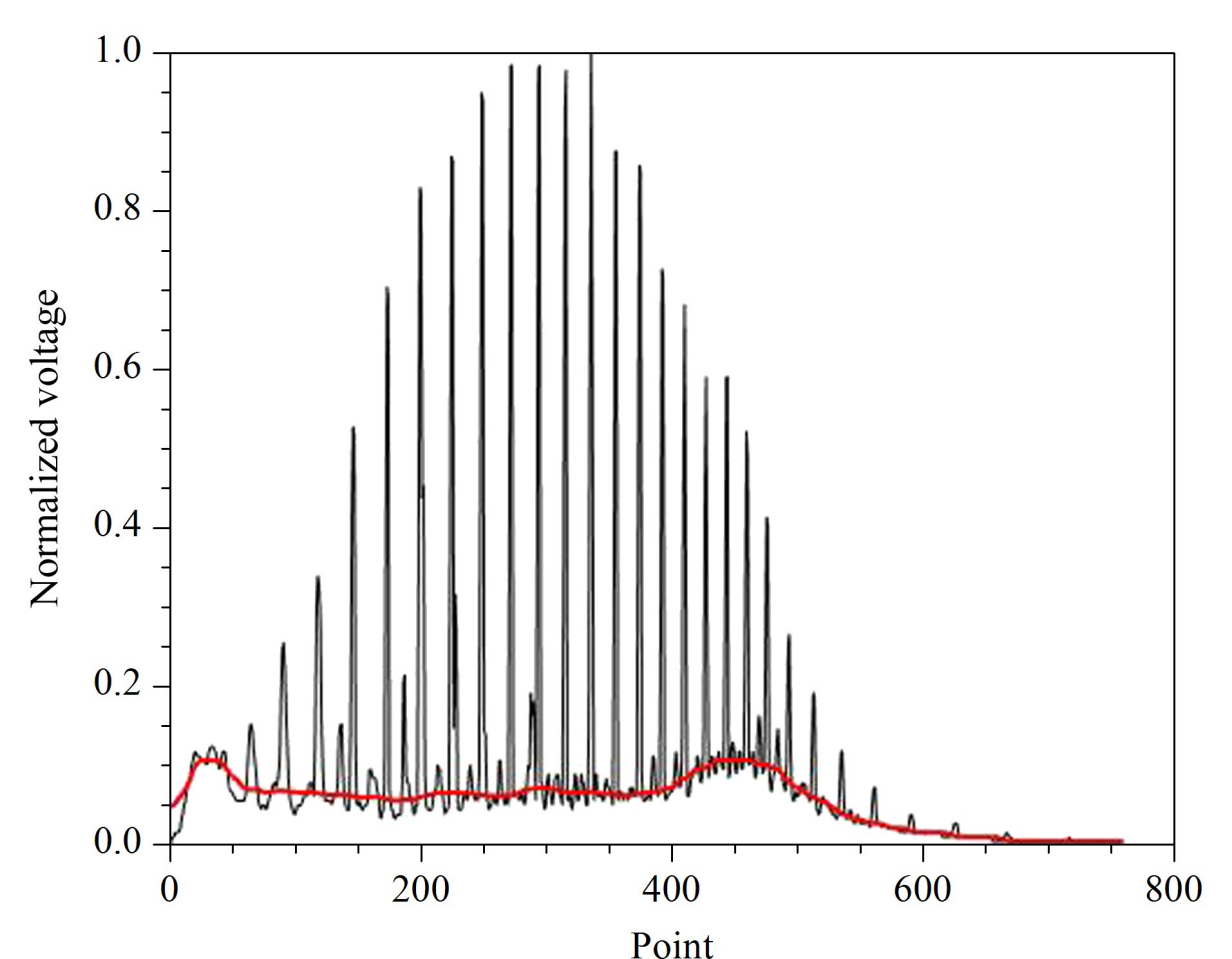
基线确定色谱图

**图5:**
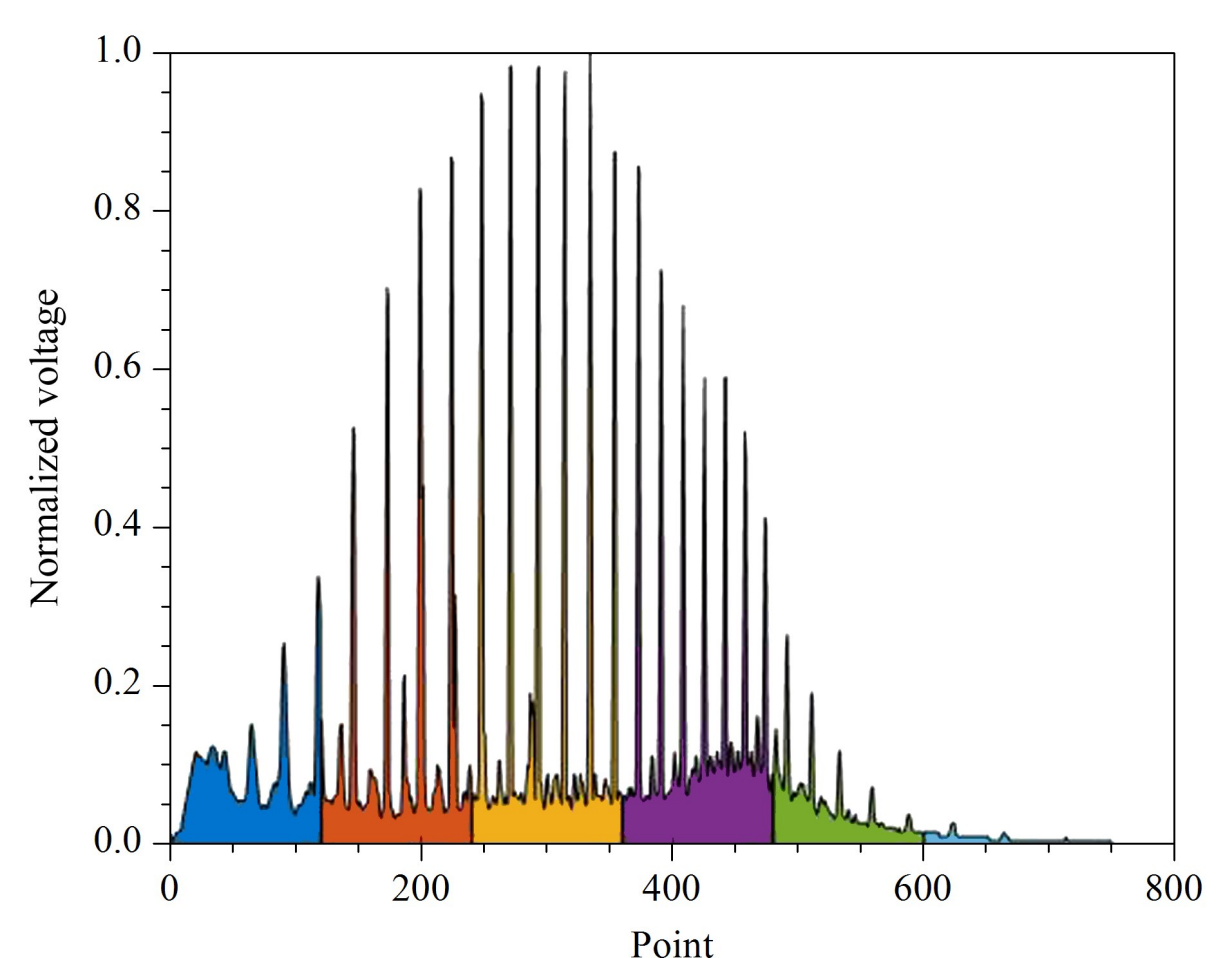
分区后的色谱图

如图5是分割后的填充色谱图,在时间轴上将色谱填充图分为6个分区,以不同颜色标记。通过计算分区面积可以得出分区面积特征与原油密度的相关性。从表1可以看出,分区顶峰连线与横轴所围绝对面积、分区基线面积、分区绝对面积相对分区矩形面积(以分区横轴为宽、纵轴为长的矩形面积)的比值与原油密度的相关性都较大。同一区域的3种分区面积特征与原油密度的相关性差距不大。因此,为了减少模型输入参数的维数,在保证6个区域所用分区面积特征是同一种的情况下,只需从3种分区面积特征中任意选取一种作为后续模型的输入参数即可,本文选择分区绝对面积作为特征参数。样本谱图的降解度和原油密度的相关系数可达0.9191,符合降解度高的样本原油密度高的规律。曲线最大峰值出现时间与原油密度相关系数为0.7092。因此本实验使用6个分区面积特征、降解度、最大峰出现时间这8个特征参数作为神经网络模型的输入。

**表1:** 各分区面积特征与原油密度的相关性

Section number of the chromatogram	Correlation coefficients		
	Absolute area	Baseline area	Ratio of partition area to partition rectangle area
1	0.2765	0.3188	0.2765
2	0.6255	0.7095	0.6255
3	0.7631	0.8233	0.7631
4	0.8912	0.9015	0.8912
5	0.7142	0.7338	0.7142
6	0.5710	0.5752	0.5710

### 2.3 实验模型评价指标

将岩屑取样谱图和壁心取样谱图各分成两组,其中83%的样本用于神经网络模型训练(100个), 17%的样本用于模型测试(20个)。利用选取的数据集分别训练建立的3种智能模型,测试结果表明,改进的广义回归神经网络模型的预测分类结果优于其他两种模型。根据前文所述储集层原油性质密度划分标准^[1]^和油气性质分类规范^[22]^,当预测密度与试油密度的绝对误差不超过0.02时,后续对原油性质的判断准确率可以达到90%以上。基于此本文将密度预测误差阈值设为0.02,各模型对岩屑取样谱图和壁心取样谱图的密度预测误差符合率如表2所示。相比于前两类预测模型,改进的广义回归神经网络模型对岩屑样本和壁心样本的预测误差符合率均在95%以上。

**表2:** 模型预测误差符合率(绝对误差<0.02)

Model	Cutting sampling/%	Wall core sampling/%
BPNN	65	75
GRNN	85	85
SSA optimized GRNN	95	100

BPNN: back propagation neural network; GRNN: generalized regression neural network; SSA optimized GRNN: generalized regression neural network optimized by sparrow search algorithm.

图6为麻雀搜索算法优化的广义回归神经网络模型迭代优化曲线,模型初次训练时适应度值较大,经过几次寻优算法的调整后,适应度值快速下降且曲线振荡较少,经过15次迭代后适应度值基本稳定在较小值,说明模型收敛速度较快且稳定,可极大地节约人工调参的时间。

**图6:**
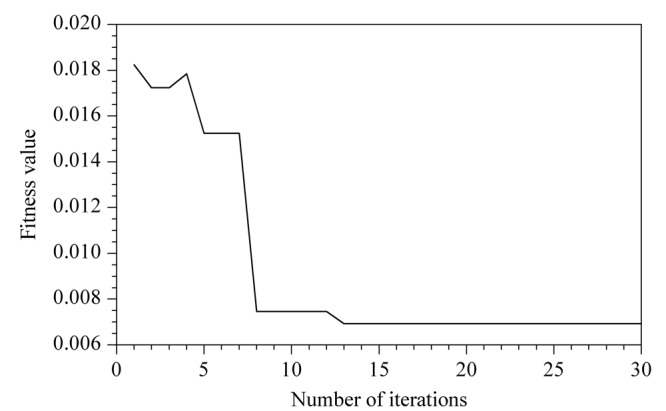
麻雀搜索算法迭代曲线

表3是各模型对密度预测的RMSE,反映了模型预测密度的离散程度和泛化能力。RMSE越小,模型对密度的预测越接近真实值,且其对不同样本谱图的预测差异性越小。图7展示了各模型对岩屑样本和壁心样本密度预测的绝对误差曲线图。结果表明,广义回归神经网络的RMSE和绝对误差均小于反向传播神经网络,且通过麻雀搜索算法优化后的广义回归神经网络对密度预测的精度和稳定性得到进一步提高。各模型预测的结果都表明,壁心取样谱图的预测效果要优于岩屑取样谱图,这说明壁心取样更能真实反应储集层原油性质。

**表3:** 模型预测的均方根误差

Model	Cutting sampling	Wall core sampling
BPNN	0.0329	0.0249
GRNN	0.0202	0.0153
SSA optimized GRNN	0.0079	0.0069

**图7:**
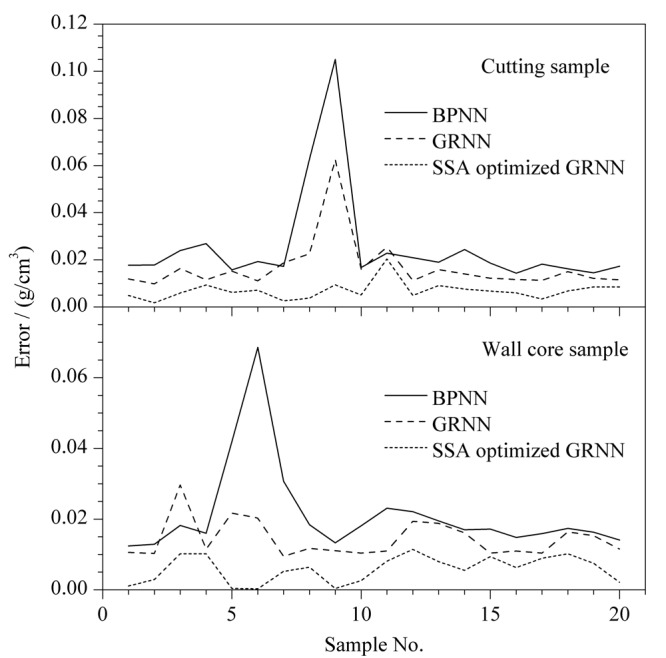
密度预测的绝对误差

根据各模型对样本谱图的密度预测结果,以及储集层原油性质密度划分规则,可以得出色谱原油性质分类结果,准确率见表4。3种模型中,预测效果最好的是改进的广义回归神经网络模型,且壁心取样获得的数据比岩屑取样获得的数据更具代表性。

**表4:** 原油性质分类准确率

Model	Cutting sampling/%	Wall core sampling/%
BPNN	85	85
GRNN	90	95
SSA optimized GRNN	95	95

## 3 结论

随着工程录井的不断发展,其对智能化的要求也越来越高。为了减少人力劳动并且更加快速准确地判断原油性质,提高效率,使用机器学习的方法对原油性质进行预测分类逐渐成为大趋势。本文针对现有的工程录井方法和原油性质判断模型的不足之处,提出了一种基于岩石热解气相色谱的储集层原油密度预测模型。对目前的岩石热解气相色谱曲线特征参数的提取方法进行了优化,通过分析各特征参数与原油密度的相关性,优选了气相色谱图中8种表征原油性质的可靠的特征参数,对比分析了反向传播神经网络、广义回归神经网络及其改进模型的效果。麻雀搜索算法优化的广义回归神经网络模型对岩屑样本和壁心样本的预测RMSE可以降到0.0079和0.0069,均优于前两种模型。与图版法、数据挖掘法相比,该模型收敛速度快,可泛化能力较强,大大减少了工作量和时间,提高了录井的效率,可为后期原油开采提供重要的数据支撑。

对于原始样本的处理方法往往决定了后期提取特征参数的真实程度,为了进一步提高模型预测的准确度,可以考虑改进样本数据处理和归一化方法,以及增加样本来源的广泛性。在本研究所建立的改进广义回归神经网络模型上,还可以进一步改进麻雀搜索优化算法,加速模型收敛速度,提高模型准确度。
